# Laparoscopic repositioning of chronic gastric volvulus in a dog

**DOI:** 10.4102/jsava.v89i0.1713

**Published:** 2018-11-06

**Authors:** Frans G. van Heerden, Marthinus J. Hartman, Vanessa McClure, Robert M. Kirberger

**Affiliations:** 1 Department of Companion Animal Clinical Studies, University of Pretoria, South Africa; 2 Onderstepoort Veterinary Academic Hospital, University of Pretoria, South Africa

## Abstract

A 12-year-old spayed Newfoundland bitch was presented with chronic non-productive vomiting, regurgitation and coughing of six weeks’ duration. On clinical examination, the dog was depressed with no other significant findings. Haematology and biochemistry investigations detected no abnormalities. Thoracic and abdominal radiographs revealed a megaoesophagus and an abnormally positioned pylorus. A thoracic and abdominal computed tomography scan confirmed the abnormal position of the stomach, together with moderate aspiration pneumonia. Laparoscopic examination of the peritoneal cavity revealed the greater omentum wrapped over the stomach, with a fold visualised between the abnormally positioned pyloric antrum and the gastric corpus. A 180-degree clockwise gastric rotation was laparoscopically diagnosed and corrected. The normal position of the stomach was confirmed before a laparoscopic-assisted incisional gastropexy was performed. Post-operatively the vomiting and regurgitation resolved and the patient was discharged. Twenty-four hours after discharge, the dog was presented with deteriorating clinical signs of aspiration pneumonia. The owner declined treatment, additional diagnostics as well as a necropsy and requested euthanasia. Chronic gastric volvulus should be considered as a rare differential diagnosis in dogs with non-specific, chronic gastrointestinal signs. Radiography, computed tomography and laparoscopy are valuable diagnostic aids in making this diagnosis. Chronic gastric volvulus can be successfully reduced laparoscopically as reported here for the first time.

## Introduction

Gastric dilatation and volvulus (GDV) occurs commonly in large, deep-chested adult dogs (Brockman, Washabau & Drobatz 1995; Green, Brown & Agnello 2012). This condition is usually acute in nature and is associated with a 10% – 43% mortality rate in patients treated for GDV (Green et al. 2012; Rawlings et al. 2002). Gastric dilatation and volvulus is defined as pyloric rotation of 90–360 degrees along the mesenteric axis of the stomach, leading to outflow obstruction and gastric dilatation. Unlike GDV, chronic gastric volvulus is uncommon in dogs (Boothe & Ackerman 1976; Frendin, Funkquist & Stavenborn 1988; Leib, Monroe & Martin 1987; Oakes & Pechman 1992; Paris et al. 2011). Dogs affected by this condition present with chronic, intermittent, non-specific clinical signs that include vomiting, anorexia and/or weight loss (Paris et al. 2011; Radlinsky 2013). Chronic gastric volvulus has been reported as a 45–180 degree clockwise or uncommonly anticlockwise rotation of the pylorus, without causing complete outflow obstruction and acute dilatation (Paris et al. 2011). This condition is dynamic, with the partial volvulus being intermittent. In a series of seven cases all but one dog responded well to surgical correction and gastropexy (Paris et al. 2011). Even though the first successful laparoscopic correction of acute GDV in two clinically affected dogs was reported by Rawlings et al. (2002), the case presented here is to the best of the authors’ knowledge the first report of laparoscopic confirmation, repositioning and gastropexy of chronic gastric volvulus.

## Ethical consideration

The Animal Ethics committee, Faculty of Veterinary Science, University of Pretoria advised that no ethical approval was required as the article is a case report. (Project ref. V083-17) The owner of the patient gave informed consent for publication.

## Case presentation

**FIGURE 1:**
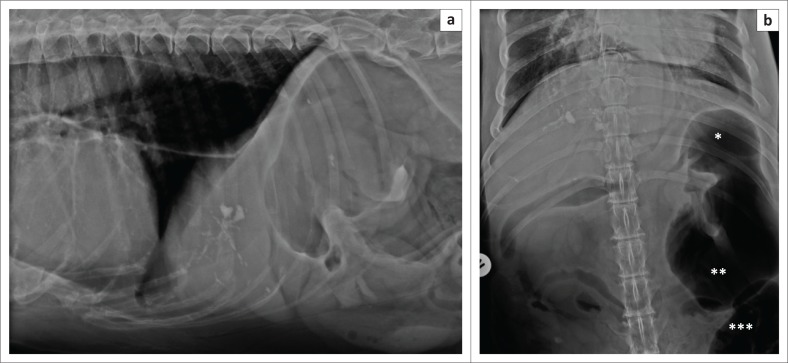
(a) Lateral caudal thorax and cranial abdominal radiograph and (b) ventrodorsal caudal thorax and cranial abdominal radiograph of a dog with chronic gastric volvulus. Note the air-filled caudal oesophagus, arborising mineralisation of the ventral liver and the abnormally positioned gas-filled stomach (a). The sagittally orientated gastric axis with the pylorus (*) cranially and fundus (**) caudally is shown in (b). The caecum and ascending colon (***) have been displaced to the left (b).

**FIGURE 2:**
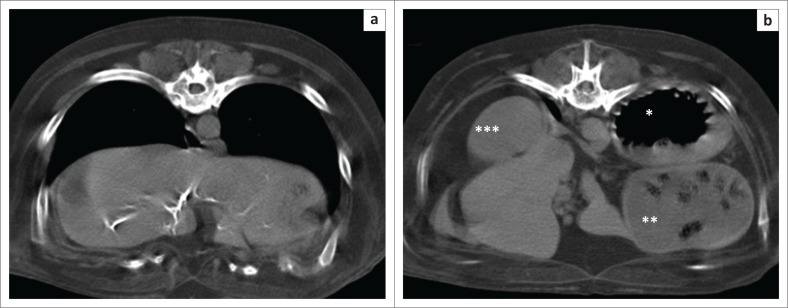
Transverse computed tomography images of the abdomen (a) at level of liver and (b) at level of stomach. Note the arborising hyperattenuating mineralised tracts, mainly in the right liver lobes in (a). In (b) the gas-filled corpus (*) is seen dorsally to the food-filled pyloric antrum (**) in the left abdomen. The kidney (***) is seen on the right side. Images are all in a soft tissue window (window width 600 HU and window level 40 HU). Left is to the right of the images. Some motion blur is present.

A 12-year-old, 66 kg, spayed Newfoundland bitch was presented with a history of non-productive vomiting, regurgitation and coughing of approximately six weeks’ duration. On clinical examination the dog was depressed with no other significant findings. Haematology, biochemistry panel, electrolytes, T4, thyroid stimulating hormone (TSH) and baseline cortisol revealed no abnormalities except for mild hyperchloraemia (118 mmol/L [reference interval 102 mmol/L – 117 mmol/L]), mild hyperkalaemia (5.5 mmol/L [reference interval 3.4 mmol/L – 5.2 mmol/L]), mild hypomagnesaemia (0.71 mmol/L [reference interval 0.77 mmol/L – 0.94 mmol/L]) and mild hyperglycaemia (6.5 mmol/L [reference interval 3.3 mmol/L – 5.5 mmol/L]). Lateral and ventrodorsal thoracic and abdominal radiographs demonstrated (Figure 1) a pneumooesophagus, which depressed the intrathoracic trachea (Figure 1a). The ascending aorta was markedly enlarged. Both kidneys were slightly smaller than the acceptable minimum size of 2.5 times the length of the second lumbar vertebra (Dennis, Kirberger & Barr 2010). The right cranial quadrant showed multiple arborising mineralised tracts in the liver. A moderately gas-distended stomach was present in the left cranial quadrant with a soft tissue band across the lumen, resulting in compartmentalisation. The pylorus was displaced cranially and to the left side, resulting in a craniocaudally orientated gastric axis (Figure 1b). The caecum and ascending colon were displaced to the mid-left abdomen, presumably secondary to the pyloric and associated descending duodenum displacement. Based on the radiographic examination, megaoesophagus, possible aortic aneurisms and kidney disease, as well as incidental choledocholithiasis were diagnosed and chronic gastric volvulus was suspected. The patient was deemed stable enough to undergo a thoracic and abdominal computed tomography (CT) scan (Figure 2), which was performed under sedation with 0.3 mg/kg butorphanol (Dolorex 10 mg/mL, Intervet, Kempton Park, South Africa). The radiological findings of chronic gastric volvulus were confirmed on CT and moderate aspiration pneumonia of the right middle lung lobe was also suspected (Figure 2b).

## Management and outcome

**FIGURE 3:**
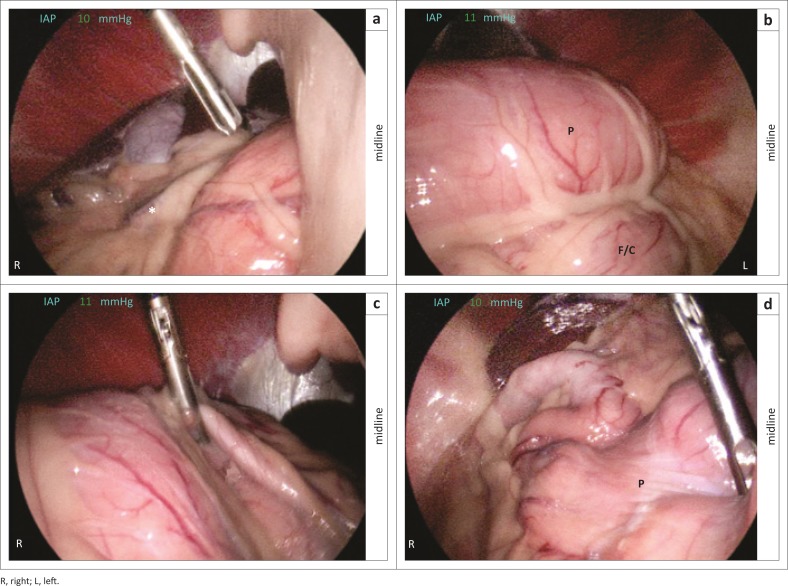
Laparoscopic image of the (a) right cranial peritoneal cavity precorrection, (b) left peritoneal cavity precorrection, (c) right peritoneal cavity and (d) image of normal position of the stomach in the right cranial peritoneal cavity after repositioning. Note the right cranial peritoneal cavity (a) illustrating the out-of-position splenic edge covered by omentum (*); the left peritoneal cavity (b) demonstrating a fold between pyloric part (P) and fundus or corpus (F/C) parts of the stomach; the right peritoneal cavity (c) demonstrating repositioning of the stomach with non-traumatic Babcock forceps; the normal position of pyloric part (P) of the stomach in the right cranial peritoneal cavity (d) after repositioning.

The megaoesophagus was thought to be a result of the chronic gastric pathology. Surgical correction of the gastric pathology was determined to be the most critical step, despite the anaesthetic risk associated with mild unilateral aspiration pneumonia. A gastroscopy performed prior to surgery was done under general anaesthesia after premedication with intravenous diazepam at 0.3 mg/kg (Pax 5 mg/mL, Pharmacare Limited, Sandton, South Africa) and induction with intravenous propofol at 4 mg/kg (propofol 20 mg/mL, Fresenius Kabi, Midrand, South Africa) to effect. Anaesthesia was maintained with isoflurane (Isofor, Safeline Pharmaceuticals, Roodepoort, South Africa). Difficulty was experienced passing the endoscope through what appeared to be a twisted lower oesophageal sphincter. Upon entering the stomach, it was found that the pyloric antrum was positioned on the left side of the patient, not in its normal position on the right. Mucosal biopsies, taken for histopathology, confirmed a *Helicobacter* infection. After gastroscopy, the patient was moved to theatre, placed on positive pressure ventilation and a 100 mm Veress needle (Veress pneumoperitoneum needle, Karl Storz Veterinary Endoscopy, Goleta, United States [US]) was used to access the peritoneal cavity. The peritoneal cavity was insufflated with CO_2_ to an intra-abdominal pressure of 13 mmHg using an insufflator (pressure-regulating mechanical insufflator, Karl Storz Veterinary Endoscopy). The Veress needle was removed and, using the same site, a 6 mm standard trocar and cannula was placed. A 5 mm, 0 degree, 29 cm laparoscopic telescope (Hopkins II telescope, Karl Storz Veterinary Endoscopy) was introduced through the cannula into the peritoneal cavity. After inspection of the peritoneal cavity, an 11 mm standard trocar and cannula was placed under direct laparoscopic visualisation on the right side of the patient, 40 mm caudal to the last rib and 40 mm lateral to the rectus abdominis muscle (Son et al. 2016). Laparoscopic examination of the cranial peritoneal cavity revealed the greater omentum wrapped over the greater curvature of the partially dilated stomach from right to left. The pyloric antrum and duodenum were not seen in their normal position in the right quadrant. The splenic edge was visualised between the liver and the stomach at this location (Figure 3a). On viewing the left cranial peritoneal cavity, a clear fold between the abnormally positioned pyloric antrum and the gastric corpus and fundus was visualised (Figure 3b). An estimated 180 degree clockwise gastric rotation was confirmed. Non-traumatic forceps (Clickline Babcock Forceps, Karl Storz Veterinary Endoscopy) were used to grasp the pyloric antrum and overlying omentum. With careful traction towards the right lateral abdomen, repetitive attempts resulted in repositioning the pyloric antrum (Figure 3c). The normal anatomic positions of the pyloric antrum, duodenum, gastric corpus and fundus, as well as adjacent spleen were confirmed after the repositioning (Figure 3d). A routine laparoscopic-assisted incisional gastropexy (LAIG) was performed (Son et al. 2016). The dog was hospitalised for four days post-operatively whilst being treated with intravenous crystalloids at 1.5 times maintenance (Ringers, Adcock Ingram Critical Care, Johannesburg, South Africa), intravenous metoclopramide (Clopamon 5 mg/mL, Pharmacare, Woodmead, South Africa) constant rate of infusion at 1 mg/kg/24 hours and subcutaneous maropitant (Cerenia 10 mg/mL, Zoetis, Sandton, South Africa) at 1 mg/kg once a day. As *Helicobacter* infection was diagnosed on the gastric biopsies it was treated with metronidazole (ADCO-Metronidazole 400 mg, Adcock Ingram Limited, Midrand, South Africa) at 10 mg/kg PO bid, amoxicillin (Betamox 500 mg, Ranbaxy Pharmaceuticals, Roodepoort, South Africa) at 20 mg/kg PO bid and omeprazole (Losec MUPS 40 mg, AstraZeneca Pharmaceuticals, Bryanston, South Africa) at 1 mg/kg PO bid (Leib, Duncan & Ward 2007). The vomiting and regurgitation resolved and the megaoesophagus was managed by feeding the patient soft food from an elevated position. The patient was kept in an upright position for 10 min post-feeding. After four days the owner insisted that the dog should be discharged as she could manage the partially resolved aspiration pneumonia on an outpatient basis. Oral antibiotics and gastroprotectants were dispensed and the owner was shown how to feed the dog appropriately. Twenty-four hours after discharge the dog was presented to our after-hours clinic with tachycardia, tachypnoea, pyrexia and abnormal lung sounds. The patient deteriorated with clinical signs related to pneumonia, but the owner declined further diagnostics and treatment and opted for humane euthanasia of the patient. A necropsy examination was declined.

## Discussion

Because of the stomach’s relatively loose suspension in the cranial abdomen by the hepatogastric ligament in the lesser omentum, it can rotate along its mesenteric axis in a clockwise or anticlockwise direction. When the rotation results in outflow obstruction, GDV typically results. Increased laxity of the hepatogastric ligament may increase stomach motility, which can predispose dogs to partial or complete gastric volvulus (Hall et al. 1995). The hepatogastric ligament and hepatoduodenal ligament might have been overlooked in the past as possible risk factors in the development of complete or partial gastric volvulus. Chronic gastric volvulus affects larger and middle-aged dogs. In the 16 cases described in the literature there were 13 large or giant breed dogs, predominantly male, with a mean age of 6 years (Boothe & Ackerman 1976; Frendin et al. 1988; Leib et al. 1987; Oakes & Pechman 1992; Paris et al. 2011). The majority of cases presented with chronic vomiting. According to recent literature, chronic gastric volvulus is also referred to as partial or chronic GDV, chronic intermittent gastric dilatation with partial volvulus and incomplete gastric volvulus (Cornell 2012; Paris et al. 2011; Radlinsky 2013). The authors suggest *chronic gastric volvulus* to be the most appropriate term for this rare condition. Multiple diagnostic modalities, including gastroscopy, are often required to diagnose chronic gastric volvulus (Paris et al. 2011). In this case radiological findings were suspicious for a chronic gastric volvulus, which was confirmed on abdominal CT. As was found in this case, chronic gastric volvulus has been described as a risk factor for the development of megaoesophagus (Gaynor, Shofer & Washabau 1997; Oakes & Pechman 1992; Paris et al. 2011). Megaoesophagus is believed to be a result of recurrent obstruction of the lower oesophageal sphincter or secondary oesophagitis caused by chronic vomition or gastroesophageal reflux (Gaynor et al. 1997). In a case series of seven dogs with chronic gastric volvulus, two dogs were diagnosed with megaoesophagus, one of which suffered from aspiration pneumonia post-operatively and was electively euthanised (Paris et al. 2011). In a separate case report, aspiration pneumonia was diagnosed in conjunction with megaoesophagus and chronic gastric volvulus (Oakes & Pechman 1992). Based on these findings, patients with chronic gastric volvulus should always be screened for megaoesophagus and aspiration pneumonia. In the case series of seven dogs with chronic gastric volvulus, five dogs had concurrent gastrointestinal disease, one of which had a low-grade *Helicobacter* spp. infection, similar to our dog (Paris et al. 2011). It is advised when chronic gastric volvulus is suspected that gastrointestinal biopsies be taken to rule out possible concurrent disease. Cases with chronic gastric volvulus may show evidence of hepatopathy resulting from chronic passive congestion of the liver, periportal fibrosis and bile duct proliferation (Guilford 1996). The cause of the cholestasis may be chronic obstruction of the common bile duct as a result of gastric displacement (Guilford 1996). In our dog, choledocholithiasis was diagnosed radiographically and on CT and may have been secondary to the chronic gastric volvulus. The abnormal gastric motility observed in chronic gastric volvulus may be an important factor in the pathogenesis of GDV (Frendin et al. 1988; Gazzola & Nelson 2014). The possibility of chronic gastric volvulus progressing to acute GDV indicates that this condition should be surgically corrected (Boothe & Ackerman 1976). Surgical correction of chronic gastric volvulus is also warranted to resolve the associated clinical signs. However, literature describing cases with chronic gastric volvulus developing into acute GDV is lacking. In a study of 44 dogs undergoing LAIG, the overall post-operative complication rate was 34%, of which 87% were minor self-limiting complications (Son et al. 2016). Reduced surgical site infection, less post-operative pain and shorter hospital stay underline the benefits of LAIG over conventional laparotomy to correct chronic gastric volvulus (Son et al. 2016).

Unfortunately, in this case neither treatment, diagnostics nor necropsy was permitted upon readmission. The authors suspect that the presenting clinical signs could have been attributed to deteriorating aspiration pneumonia.

## Conclusion

Chronic gastric volvulus should be considered as a rare differential diagnosis in dogs that present with non-specific, chronic gastrointestinal signs. Radiography, CT and laparoscopy are valuable diagnostic aids in making this diagnosis. Chronic gastric volvulus can be successfully reduced laparoscopically and stabilised with a minimally invasive LAIG procedure.
